# Supplementary material for: Assessing the Clinical Relevance of Soluble PD-1 and PD-L1: A Multi-Cohort Study Across Diverse Tumor Types and Prognostic Implications
Source: Biomedicines. 2025 Feb 17;13(2):500. doi: 10.3390/biomedicines13020500 (PMC11852959; doi:10.3390/biomedicines13020500)
Supplement: Supplementary file 1 [file biomedicines-13-00500-s001.zip › biomedicines-3429068-supplementary.pdf]

Characteristic	No of patients	%
Breast cancer		
Median age (range), years	49 (30-72)	
Histology		
Luminal	40	60
HER2+	5	7
TNBC	22	33
Stage		
II	20	30
III	47	70
Tumor size		
T1-T2	29	43
T3-T4	38	57
Nodal status		
N0	16	24
N+	51	76
Grade		
G2	50	75
G3	17	25
Ovarian cancer		
Median age (range), years	55 (29-78)	
Histology		
Serous	81	79
Mucinous	8	8
Endometrioid	13	13
Stage		
I-II	37	36
III-IV	65	64
Tumor size		
T1-T2	39	38
T3-T4	63	62
Nodal status		
N0	93	91
N+	9	9
Metastasis		
M0	95	93
M+	7	7
Grade		
G1-G2	45	44
G3	57	56
Renal cancer		
Median age (range), years	60 (33-81)	

Gender		
male	63	60
female	42	40
Histology		
ccRCC	90	86
nccRCC	15	14
Stage		
I-II	67	64
III-IV	38	36
Tumor size		
T1-T2	74	70
T3-T4	31	30
Nodal status		
N0	90	86
N+	15	14
Metastasis		
M0	92	88
M+	13	22
Grade (without chRCC)		
G1-G2	67	71
G3-G4	28	29
Bone cancer		
Median age (range), years	40 (18-76)	
Gender		
male	69	61
female	44	39
Histology		
Osteosarcoma	37	33
Chondrosarcoma	55	49
Ewing’s sarcoma	8	7
Chordoma	13	11
Stage		
I	18	16
II	77	68
III-IV	18	16
Tumor size		
T1	17	15
T2	83	73
T3	13	12
Nodal status		
N0	110	97
N+	3	3
Metastasis		

M0	106	94
M+	7	6
Grade		
G1-G2	46	41
G3	67	59
Gastric cancer		
Median age (range), years	60 (25-81)	
Gender		
male	58	57
female	44	43
Histology		
Adenocarcinoma	78	77
Signet ring cell carcinoma	23	23
Stage		
I-II	39	39
III-IV	62	61
Tumor size		
T1-T2	22	22
T3-T4	79	78
Nodal status		
N0	48	48
N+	53	52
Metastasis		
M0	77	76
M+	24	24
Grade		
G1-G2	27	27
G3	74	73
Colorectal cancer		
Median age (range), years	62 (27-85)	
Gender		
male	144	54
female	123	46
Localization		
Left	210	79
Right	57	21
Stage		
I-II	153	57
III-IV	114	43
Tumor size		
T1-T2	63	24
T3-T4	204	76
Nodal status		

N0	165	62
N+	102	38
Metastasis		
M0	221	83
M+	46	17
Grade		
G1-G2	71	27
G3	196	73
